# Acute Ischemic Stroke Superimposed on Acute Pulmonary Embolism in the Presence of Patent Foramen Ovale: A Case Report

**DOI:** 10.7759/cureus.61036

**Published:** 2024-05-25

**Authors:** Khaled Alaboud Alkheder, Yousif Basim Al-Khafaji, Omar Farooq Al-Nahhas

**Affiliations:** 1 Emergency Medicine, Tawam Hospital, Al-Ain, ARE

**Keywords:** venous thromboembolism, paradoxical embolism, anticoagulation, congenital heart disease, stroke, atrial septal aneurysm, pulmonary embolism, patent forman ovale (pfo)

## Abstract

A patent foramen ovale (PFO) carries a high risk of paradoxical embolism. This risk is higher in certain conditions, including acute pulmonary embolism (APE). Although most patients with a PFO are asymptomatic, various clinical manifestations may be associated with PFO. Concomitant APE and acute ischemic stroke (AIS) due to paradoxical embolism from a PFO are rare. We report a case of a 61-year-old man who presented with simultaneous PE and AIS in the presence of PFO, was treated successfully with anticoagulation, and was discharged from the hospital neurologically intact.

## Introduction

The presence of a patent foramen ovale (PFO) escalates the risk of acute ischemic stroke (AIS) in those who coincidentally present with pulmonary embolism (PE). This risk increases dramatically, especially in those with PFO [1]. The management of coexisting ischemic stroke and PE presents a formidable challenge to the healthcare team due to conflicting opinions and the absence of clear guidelines [2].

## Case presentation

We describe a 61-year-old man with no known past medical history who was transferred to our Emergency Department (ED) due to sudden loss of consciousness and shortness of breath preceded by right-sided weakness. In further history by his accompanying colleague, he mentioned that the patient was on the bus when he experienced weakness, could not move his right upper limb, and had an altered vision where he saw “black curtains falling.” He denied having headaches, changes in speech, abnormal jerky movements, chest pain, or lower limb weakness. After this episode, he started having dyspnea and lost consciousness for an unknown duration before coming to the ED. Furthermore, he was vitally stable in the ED and started gaining consciousness; a physical exam was done and was normal. Subsequently, the stroke code was activated.

**Figure 1:**
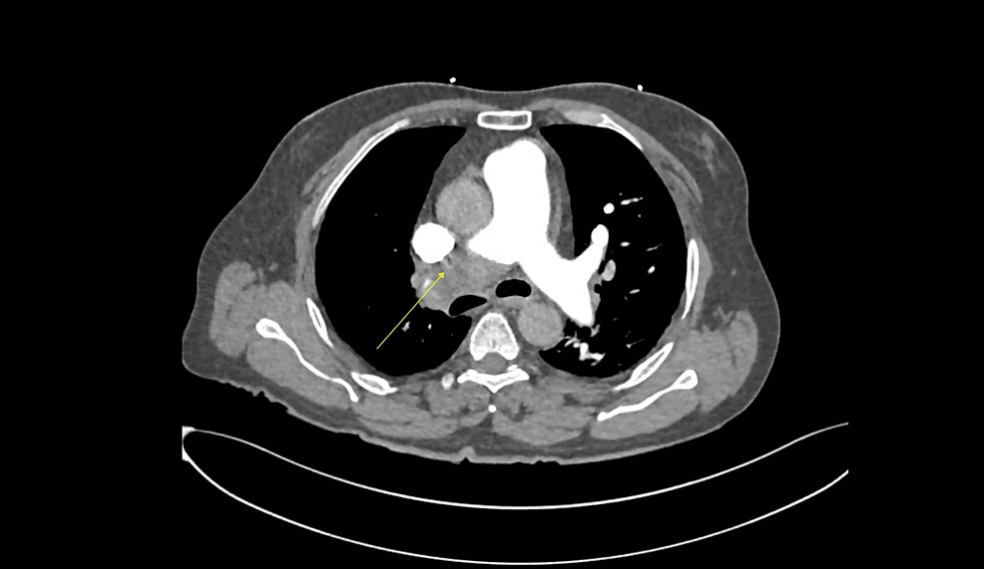
Right pulmonary trunk showing the filling defect

**Figure 2:**
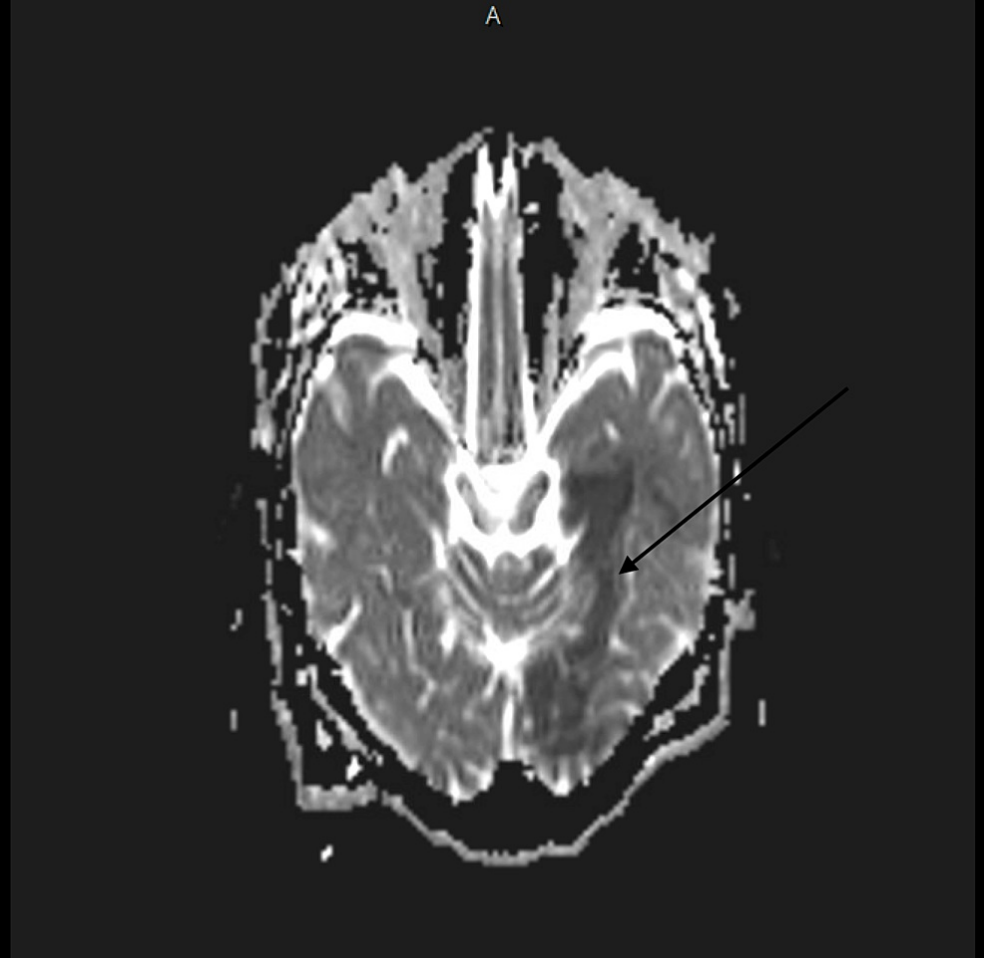
ADC MRI scan of the brain delineating the affected parts ADC, apparent diffusion coefficient

**Figure 3:**
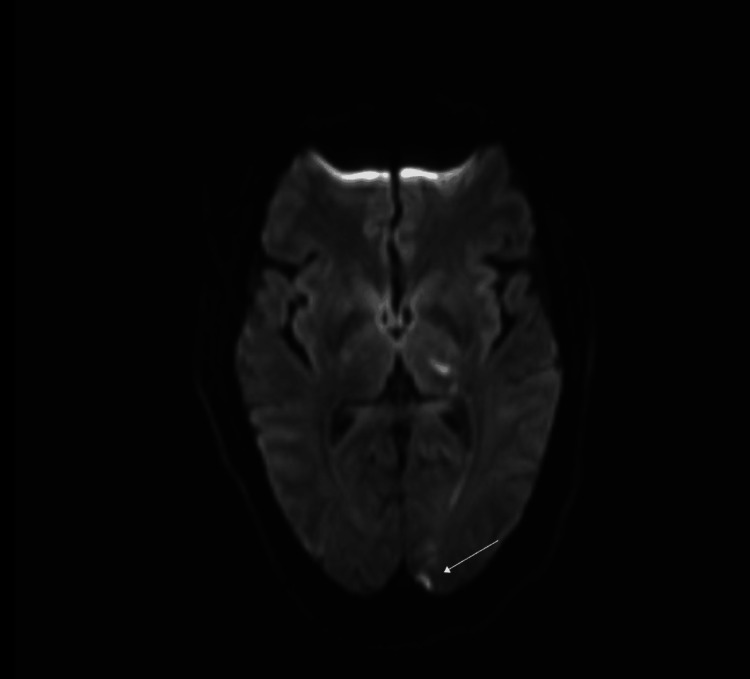
Diffusion-weighted MRI of the brain showing an area of diffusion restriction on the left occipital lobe

**Figure 4:**
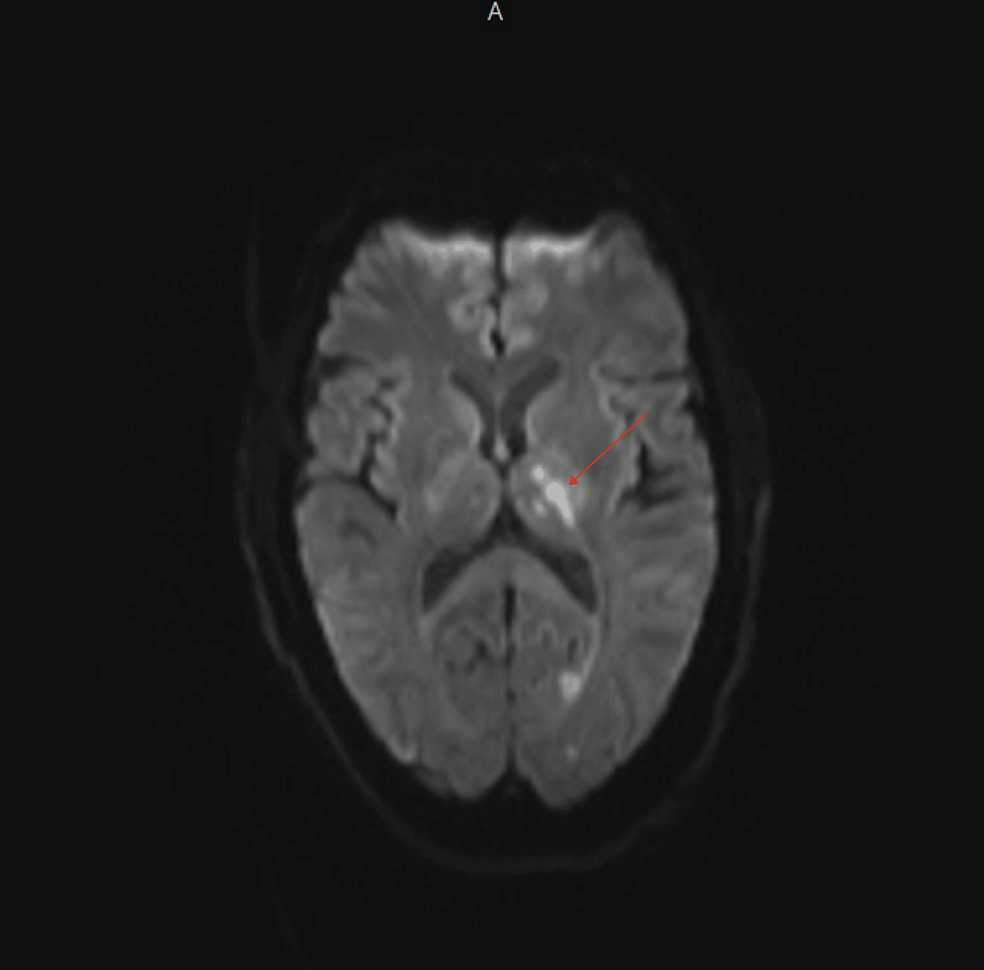
Diffusion-weighted MRI of the brain showing an area of diffusion restriction at the junction of the left thalamus and posterior limb of the internal capsule

Plain and contrast-enhanced computed tomography (CT) of the head and neck was done and reported an unremarkable origin of great vessels, carotids, and vertebral arteries, in addition to a normal appearance of the middle cerebral arteries (MCAs), posterior inferior cerebellar arteries (PICAs), posterior cerebral arteries (PCAs), and the anterior cerebral arteries (ACAs). Interestingly, incidental findings of suspected thrombus in the right pulmonary artery were noticed. Consequently, contrast-enhanced chest CT was done and revealed a filling defect in the superior part of the right pulmonary artery and its branches and subsequent lack of visualization of the right upper pole of the lung's vasculature, which most likely signifies PE (Figure 1). Subsequent brain MRI findings showed left anterior choroidal and left PCAs territory stroke, which are consistent with showering embolic stroke (Figures 2-4).

**Figure 5:**
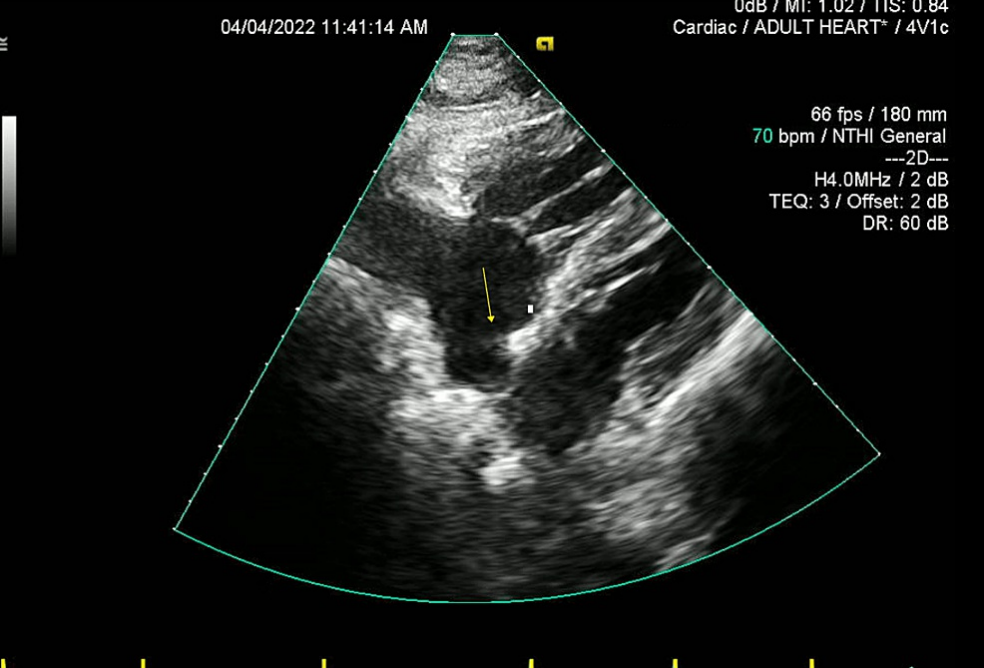
Transthoracic ECHO delineating the aneurysmal inter-atrial septum

As part of the stroke workup, transthoracic and transesophageal echocardiography (TEE) were done and showed features of the aneurysmal interatrial septum (ASA); the presence of PFO was confirmed by the bubble test on TEE, along with a right-to-left shunt and dilated right atrium and ventricle along with severely reduced ventricular function (Figure 5). Also, prothrombotic screening (including, protein C and S, antithrombin III, and anticardiolipin) was done and all were negative. Finally, the bilateral lower limbs Doppler study was negative for deep vein thrombosis as well. 

After discussion with the neurology, cardiology, and pulmonology teams, the decision was taken to start the patient on therapeutic anticoagulation along with a single antiplatelet and to admit the patient for further management. Hence, he was started on a therapeutic dose of Lowe molecular-weight heparin (LMWH) along with aspirin. Since the patient was vitally stable throughout his hospital stay and his Pulmonary Embolism Severity Index (PESI) score was 70, there was no clear indication to thrombolyse him. The cardiology team concluded that there is no urgency for surgical closure of his PFO at the moment, and he can be booked later after his discharge for the procedure. The patient was then referred to another center with expertise for surgical closure of the PFO and was counseled about the need to continue the treatment with direct oral anticoagulant (DOAC), namely, edoxaban for six months, and to be followed up after that. Fortunately, the patient was discharged neurologically intact and in good health.

## Discussion

The presence of PFO increases the risk for AIS in patients presenting with PE, in fact, the frequency could be quadrupled in those with PFO [1]. In addition, the presence of ASA would play a role in increasing the risk for recent ischemic stroke in those with PFO [2,3]. Our patient’s echocardiographic finding was consistent with the presence of both ASA and PFO, which might have explained his increased risk of stroke that led to its occurrence, especially in the presence of PE.

Management of coexisting ischemic stroke and PE poses a challenge to the caring team given the contradicting opinions and the lack of clear guidelines. The dilemma arises from the fact that the management of massive PE is usually by thrombolysis which at the same time is absolutely contraindicated in ischemic stroke within three months [4]. On the other hand, intravenous thrombolysis is the recommended treatment for patients presenting with acute stroke within three hours of onset (class I, level of evidence A) [5]. Also, there is a high possibility of hemorrhagic transformation of an ischemic stroke if PE is managed by therapeutic heparin and that adds to the controversy. A prospective study by Goliszek et al. included 55 patients with a median age of 49 years where 5.5% were diagnosed with high-risk PE. While 52 patients were hemodynamically stable initially, 29% showed right ventricular dysfunction (RVD), forming an intermediate-risk acute PE group, and 65.5% had low-risk acute PE; their observations suggest that even clinically silent acute ischemic stroke may increase the risk of intracranial hemorrhage [6]. In our patient, we opted for therapeutic anticoagulation using LMWH during his hospital stay. The option of thrombolysis was not considered given the unknown time for the onset of his stroke and the absence of high-risk PE or signs of instability. Another important factor to be taken into consideration is the prevention of paradoxical embolism in those with PFO, which is a topic still being discussed in the literature [7]. Despite the anticoagulation and PFO closure being the mainstay for secondary stroke prevention, mounting evidence recommends PFO closure over anticoagulation as an effective strategy to prevent paradoxical embolism; however, it carries a risk for atrial fibrillation [8,9]. 

## Conclusions

In conclusion, the presence of PFO in patients with PE significantly heightens the risk of AIS, especially in the presence of ASA. Managing coexisting AIS and PE poses a clinical dilemma due to conflicting expert opinions and a lack of uniform guidelines. In our patient, therapeutic anticoagulation with LMWH was chosen over thrombolysis, given the absence of high-risk PE features or signs of instability. However, the ongoing debate surrounding PFO closure versus anticoagulation for preventing paradoxical embolism underscores the need for individualized treatment strategies. Further research is warranted to elucidate the optimal approach for managing this complex clinical scenario.
